# Effect of virtual reality combined with repetitive transcranial magnetic stimulation on musculoskeletal pain and motor development in children with spastic cerebral palsy: a protocol for a randomized controlled clinical trial

**DOI:** 10.1186/s12883-023-03359-4

**Published:** 2023-09-26

**Authors:** Xin Li, Zefan Huang, Tijiang Lu, Juping Liang, Haibin Guo, Lixia Wang, Zhengquan Chen, Xuan Zhou, Qing Du

**Affiliations:** 1 Department of Rehabilitation Medicine, Xinhua Hospital, School of Medicine, Shanghai Jiao Tong University, 1665 Kongjiang Road, Shanghai, 200092 China; 2 School of Exercise and Health, Shanghai University of Sport, Shanghai, China; 3 Xinhua Hospital, School of Medicine, Shanghai Jiao Tong University, Shanghai, China; 4 Chongming Hospital, Shanghai University of Medicine & Health Sciences, Shanghai, China

**Keywords:** Cerebral palsy, Virtual reality, Pain, Repetitive transcranial magnetic stimulation, Randomized Controlled Trial, Protocol

## Abstract

**Purpose:**

This trial aims to investigate the efficacy and safety of virtual reality (VR) combined with repetitive transcranial magnetic stimulation (rTMS) for improving musculoskeletal pain and motor development in children with unilateral spastic cerebral palsy (CP).

**Methods:**

This study protocol is for a randomized controlled trial consisting of 2 treatment sessions (3 days/week for 4 weeks in each session, with a 1-week interval between sessions). We will recruit children aged 3–10 years with unilateral spastic CP (Gross Motor Function Classification System level I or II). Participants will be randomly divided into 3 groups: the VR + rTMS group (immersive VR intervention, rTMS and routine rehabilitation therapy), rTMS group (rTMS and routine rehabilitation therapy), and control group (sham rTMS and routine rehabilitation therapy). VR therapy will involve a daily 40-minute movement training session in a fully immersive environment. rTMS will be applied at 1 Hz over the primary motor cortex for 20 min on the contralateral side. The stimulation intensity will be set at 90% of the resting motor threshold, with 1200 pulses applied. A daily 60-minute routine rehabilitation therapy session including motor training and training in activities of daily living will be administered to all participants. The primary outcome will be pain intensity, assessed by the Revised Face, Legs, Activity, Cry, and Consolability Scale (R-FLACC). The secondary outcomes will include motor development, evaluated by the 66-item version of the Gross Motor Function Measure (GMFM-66) and Fine Motor Function Measure (FMFM); balance capacity, measured by the interactive balance system; activities of daily living; and quality of life, measured by the Barthel index and the Chinese version of the Cerebral Palsy Quality of Life scale for Children (C-CP QOL-Child). Safety will be monitored, and adverse events will be recorded during and after treatment.

**Discussion:**

Combined application of VR therapy and rTMS may reveal additive effects on pain management and motor development in children with spastic CP, but further high-quality research is needed. The results of this trial may indicate whether VR therapy combined with rTMS achieves a better analgesic effect and improves the motor development of children with spastic CP.

**Trial registration:**

Registration number: ChiCTR230069853. Trial registration date: 28 March 2023. Prospectively registered.

## Introduction

Cerebral palsy (CP) is “a disorder of movement and posture due to a defect or lesion of the immature brain” [1, 2]. Pain is one of the most cited musculoskeletal disorders in spastic CP children, with a prevalence of up to 74% [3, 4]. It is an urgent issue in spastic CP, as pain may trigger physical and emotional complications, such as decreased mobility and irritability [5]. Chronic pain in children with spastic CP remains consistent (45%) or worsens (34%) between clinical visits and is considered strongly associated with reduced quality of life, as it may impact exercise and social participation, leading to developmental delay of motor and language function, as well imposing an enormous personal and economic burden [6].

Chronic primary pain is associated with injury to the thalamus or spinothalamic tract [7]. Primary pain from a musculoskeletal origin has rarely been reported [8]. Secondary pain caused by muscle spasms, postural asymmetries, and osteoarthrosis complications is highly prevalent in spastic CP. Muscle spasms lead to vessel compression and ischaemia, which activate local pain receptors [9]. Soft tissue may be under long-term compression due to postural asymmetries, and the resulting chronic pain occurs mostly in the back [10]. However, the identification of pain is often difficult for children who experience pain because children with spastic CP may not accurately express pain due to cognitive limitations [11]. Numerous previous studies have suggested that physical and occupational therapy may be basic treatments for pain control, including music therapy [12], games [13], and deep breathing training [14]. However, in a substantial proportion of CP patients, pain remains refractory and requires further therapy [3]. To date, analgesic-based treatments have focused only on pain and physical symptoms, neglecting overall function, especially balance function and activities of daily living.

Virtual reality (VR), a new technology for implementing innovative rehabilitation treatments in cognitive and motor domains [15], provides people with multisensory stimulation without nociception and has been proven to be effective in relieving chronic pain by distracting patients, diverting attention, and enabling them to develop skills that modulate pain processing [16]. Moreover, the virtual tasks provided by VR therapy may allow people to complete directed body movements in specific scenes, such as indoor or natural environments, accompanied by visual, auditory, and tactile feedback [17]. Clinical trials have shown that VR therapy can reduce inflammation in chronic low back pain [18, 19] and has been widely used in adults with spinal cord injury, brain trauma, and stroke to provide analgesia [20, 21]. In addition, VR therapy has the advantage of enhancing cognitive and motor function by stimulating multiple sensory systems, and it has been proven to be effective in relieving burn and dental pain in children [17]. VR therapy combined with conventional therapeutic exercise for children with CP can promote their engagement in rehabilitation therapy, increasing interest and motivation [22, 23], but more evidence is needed to determine the effect of VR therapy on pain management in CP patients.

Emerging evidence supports the use of repetitive transcranial magnetic stimulation (rTMS) to treat pain and headache by stimulating either the primary motor cortex (M1) or dorsolateral prefrontal cortex (DLPFC) [24, 25]; this method has also been widely used to relieve pain in patients with chronic diseases, including Parkinson’s disease, fibromyalgia and intractable postherpetic neuralgia [26–28], by regulating the membrane potential of central neurons through pulsed magnetic fields and mediating the excitability of central neurons to affect the electrical activity of the central nervous system [24]. As noninvasive and painless nerve regulation technologies, both high frequency (≥ 5 Hz, used to decrease excitability) and low frequency (≤ 1 Hz, used to increase excitability) rTMS of the M1 or primary visual cortex have been proven to be safe therapies for children with at intensities of 130% of the resting motor threshold [29, 30], and long-term follow-up showed that there was a cumulative effect leading to continuous pain relief from 7 or more rTMS sessions [31].

Despite the acceptability of VR in children and the sustained effects of rTMS, it is currently unknown whether there is a synergistic effect of the combination of VR therapy and rTMS on pain in children with spastic CP. This study protocol combined VR therapy and rTMS with the aim of exploring the efficacy and safety of this combined treatment in improving the pain and motor development of children with spastic CP. We hypothesize that rTMS can improve pain in children with CP and that the combination of VR therapy and rTMS will show better efficacy than rTMS alone.

## Methods

### Study design

**Fig. 1:**
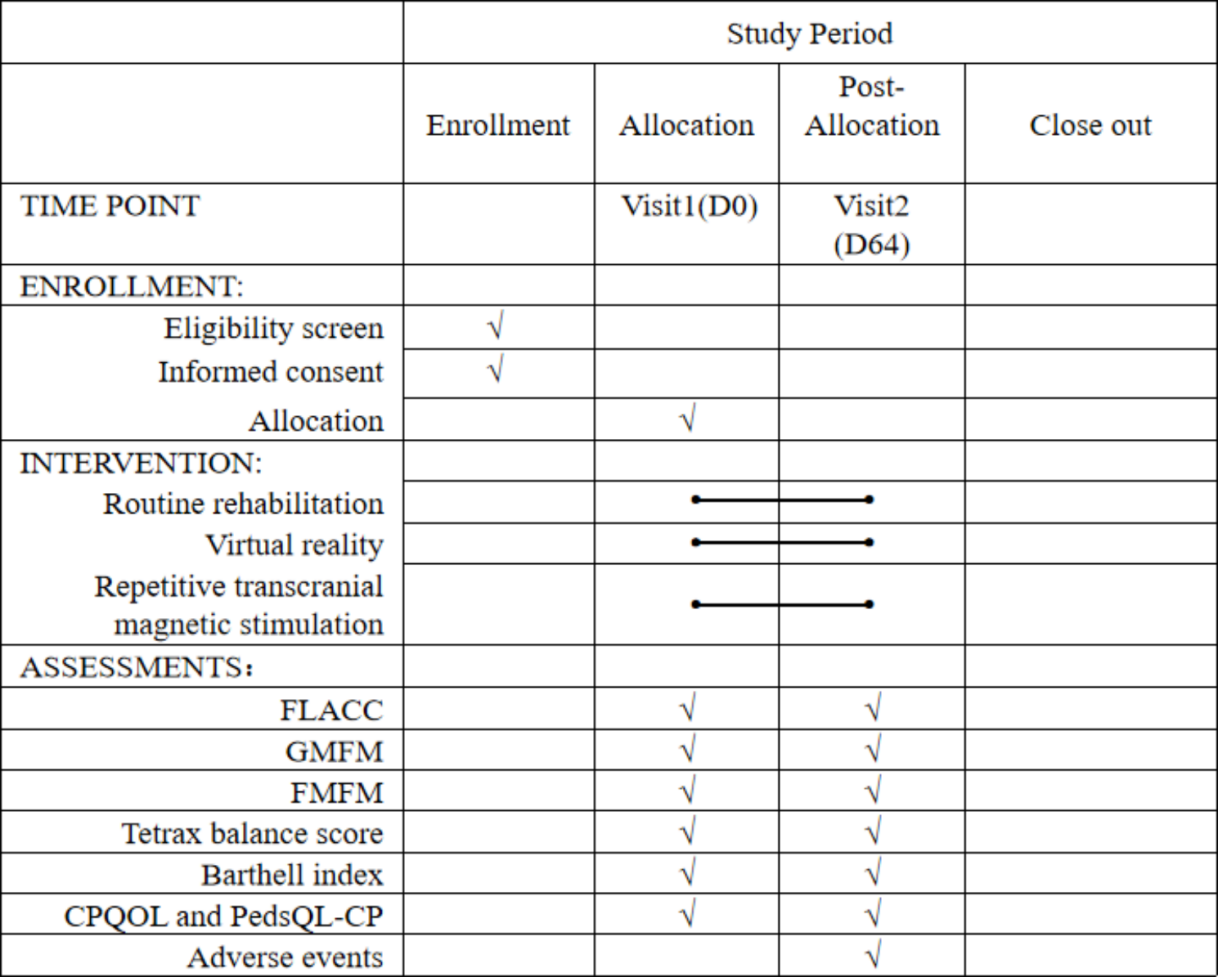
SPIRIT checklist. FLACC, Face, Legs, Activity, Cry, and Consolability Scale; GMFM-66, 66-item version of the Gross Motor Function Measure; FMFM, Fine Motor Function Measure; C-CPQOL-Child, Chinese version of the Cerebral Palsy Quality of Life scale for children; PedsQL-CP, Pediatric Quality of Life Inventory Measurement Model: Cerebral Palsy Module

**Fig. 2:**
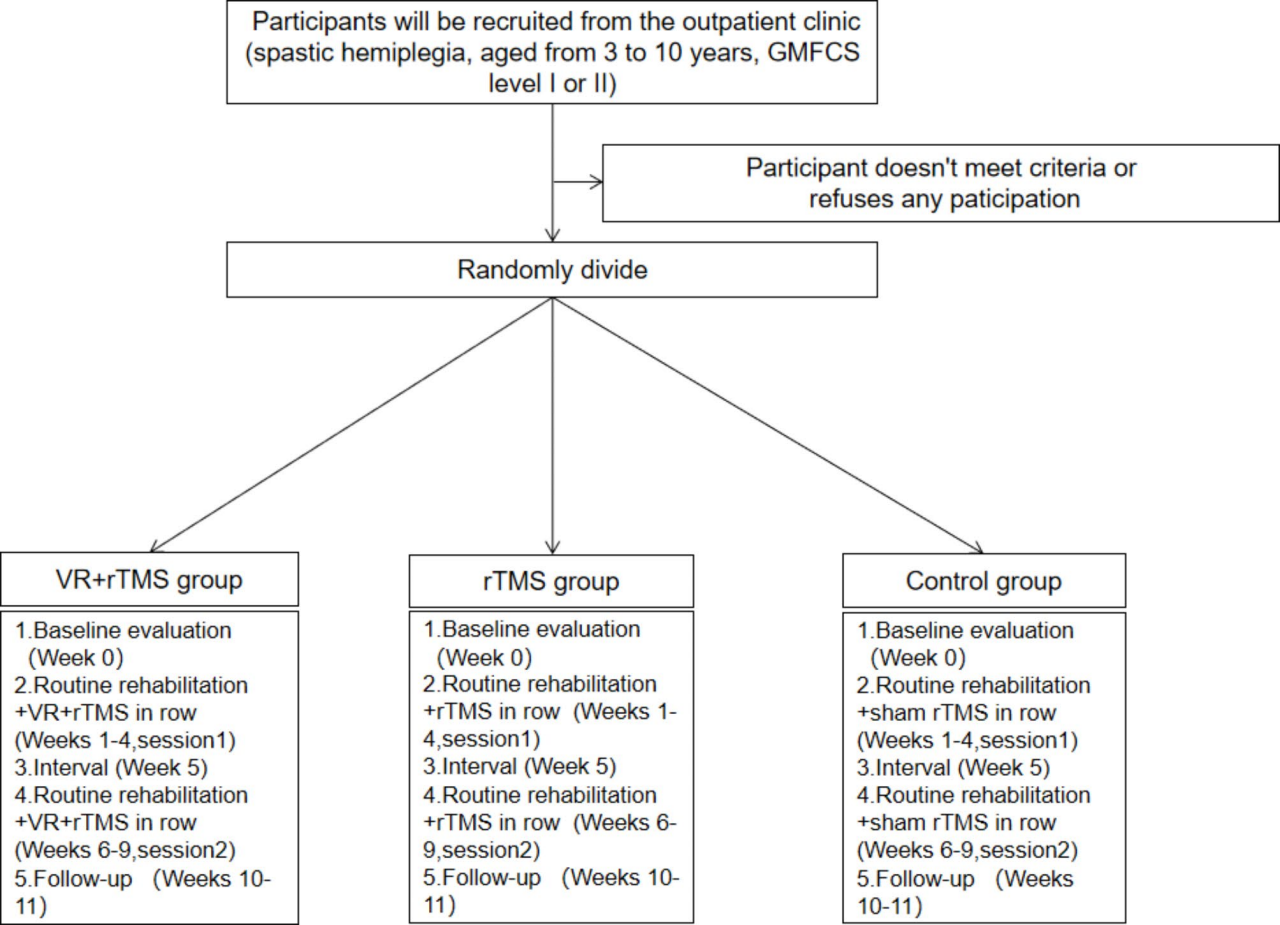
CONSORT flowchart. GMFCS, Gross Motor Function Classification System; VR, virtual reality; rTMS, repetitive transcranial magnetic stimulation

This is a randomized, single-blind (evaluator), prospective clinical trial (protocol version 2.0, date: 2023.02.28). Participants will be randomly and equally divided into 3 groups: the VR + rTMS group, the rTMS group, and the control group. Two 4-week sessions of treatment will be provided to participants, separated by an interval of one week. Assessments will be performed at baseline (week 0) and after the intervention (week 9). This study will follow the Standard Protocol Items: Recommendations for Intervention Trials (SPIRIT) guidelines [32] (Fig. 1) and the Consolidated Standards of Reporting Trials (CONSORT) statement (http://www.consort-statement.org (Fig. 2) [33]. The study’s trial registration number is ChiCTR230069853.

### Ethics

The procedures in this protocol are in accordance with the standards of the Declaration of Helsinki [34]. The trial will adhere to the study protocol. The private and personally identifiable information of the participants will be strictly protected. This study has been approved by the Xinhua Hospital Ethics Committee (Shanghai, China) [XHEC-C-2023-006-2]. Two sets of comparisons will be conducted (the VR + rTMS group vs. the rTMS group and the rTMS group vs. the control group). Considering a potential dropout rate of 15%, a total of 108 children (36 in each group) will be enrolled. Written informed consent to participate will be obtained from all study participants and in case of minor’s informed consent will be obtained from parents/legal guardian(s) before the children are randomly assigned to groups and can choose the desired treatments after the trial.

### Study population, recruitment, and eligibility criteria

Assessment will be undertaken by the same physiotherapy team experienced in the management of paediatric patients. All assessors involved in data collection will be trained in the study procedures and familiarized with the use of the study instruments and measurements. Participants will volunteer to participate through the outpatient service of the Rehabilitation Medicine Department, Xinhua Hospital, School of Medicine, Shanghai Jiaotong University. Children who are diagnosed with CP by an experienced paediatrician based on the Chinese Rehabilitation Guidelines for Cerebral Palsy (2022) [35] will be included. Another trained paediatrician will provide information about this study both verbally and in writing to children who meet the eligibility criteria. Children and their guardians will be informed that they will be randomly assigned to one of three effective treatments to determine which treatment best improves pain and development. After enrolment, an independent researcher will implement random assignment by telephone to avoid predictability. Basic information (age, sex, height, weight) and function classification with the Gross Motor Function Classification System (GMFCS) will be collected [36].

#### Inclusion criteria

① Diagnosis of spastic CP,
② Aged 3 to 10 years,
③ GMFCS level I or II,
④ A history of pain reported by participants or their guardians.

#### Exclusion criteria

① A history of brain trauma or brain surgery;
② Received any drugs, surgery, or rehabilitation training to improve CP symptoms in the past 6 months;
③ Contraindications for rTMS, including cochlear or intracranial implants, elevated intracranial pressure, intracranial infection, and acute haemorrhagic diseases;
④ Inability to cooperate with VR treatment due to severe cognitive, visual, or hearing impairment.

### Random assignment and blinding

Random assignment will be performed based on the patient number and a random number sequence. The physiatrist in charge of patient recruitment will number patients according to the registration order. An independent researcher will generate a random number sequence (random seed: 16,264) through a website (www.randomization.com) and seal the allocation result in 108 sequentially numbered envelopes. Chief physiotherapists for the intervention will individually obtain the random allocation results to confirm the interventions. We will prepare a case report form for each participant to record evaluation results. Outcome measurements will be collected at baseline and after the intervention in a specialized venue by one experienced physician trained in basic and advanced courses for paediatric functional assessment. This assessor will be employed full time and will not participate in the design and group allocation of this study and will be unaware of the study objectives and the group allocation of patients; this assessor will not have access to the random allocation sequence. Moreover, he or she will be blinded during the entire process (including blinded to patient names, outpatient numbers, and other important information related to the identity of the patients) and will not be able to obtain relevant information on group allocation and intervention methods.

### Sample size

G*Power 3.1 software was used to calculate the sample size. Based on Sahin [37] et al., the effect size was set to 0.9, with α = 0.025 and β = 0.1. Two sets of comparisons will be conducted (the VR + rTMS group vs. the rTMS group and the rTMS group vs. the control group). Considering a potential dropout rate of 15%, a total of 147 children (49 in each group) will be enrolled in this study.

### Intervention

The participants will receive 2 treatment sessions (4 weeks/session, 3 days/week). There will be a one-week interval between the 2 treatment sessions.

#### VR + rTMS group

Participants in the VR + rTMS group will receive VR therapy, rTMS, and routine rehabilitation. The interventions will be conducted in the following order: routine rehabilitation, VR therapy, and rTMS.

The virtual reality rehabilitation system (VRRS, Khymeia Group, Noventa Padovana, Italy) will be used to administer VR therapy. Each participant will be fully immersed in the virtual environment that can provide visual and acoustic feedback. Participants will be guided to perform active motions such as touching or grasping specific objects, balance control, lower limb movement, and hip flexion in specific situations for 40 min [38]. Before the start, therapists will adjust the difficulty and intensity according to the child’s motor function and responses to VR.

rTMS will be administered through a figure-eight coil connected to a Yiruide NS1000 stimulator (Yiruide Company Limited, Wuhan, China). The resting motor threshold (rMT) is defined as the stimulation intensity that induces a motor evoked potential of the contralateral abductor pollicis brevis that reaches 50 µV more than 5 times in 10 stimulations applied to the primary motor cortex (M1). The rMT will be measured in every child before each course of treatment. LF-rTMS (1 Hz) will be applied to the M1 for 20 min on the unaffected side. The stimulation intensity will be set at 90% rMT, with a total number of pulses of 1200 [39].

Routine rehabilitation consists of movement therapy and occupational therapy. The participants will receive 40 min of exercise training every day mainly on controlling the head, rolling over, sitting, crawling, standing, and walking. After movement therapy, participants will complete 20-minute occupational training tasks, mainly including upper limb function training, sensory integration therapy, and orthotics usage.

#### rTMS group

Both the routine rehabilitation treatment and TMS for children in the rTMS group will be the same as those of the VR + rTMS group.

#### Control group

The control group will receive the same routine rehabilitation regimen as the rTMS group, but a 20-minute sham stimulation will replace rTMS after routine rehabilitation therapy. A figure-eight advanced sham coil will be located on the unaffected side over the M1. The appearance, operation process, sound, and vibration sensation of the device during treatment will be the same as that during verum rTMS [40].

### Outcome measure

#### Primary outcome

The primary outcome will be assessed at baseline and after the intervention. Pain measured by the revised FLACC scale (R-FLACC) [41] will be the primary outcome. With this scale, the physiatrist will observe the child’s uncovered body and legs for at least five minutes, describing the child’s pain level from 5 dimensions (Face, Legs, Activity, Cry, Consolability) and then provide scores on a three-point scale (ranging from 0 to 2) for each dimension. Higher scores indicate a higher pain level. The total score of the R-FLACC will be analysed.

#### Secondary outcome

Secondary outcomes will be assessed at baseline and after the intervention.

The 66-item version of the Gross Motor Function Measure (GMFM-66) [42] and Fine Motor Function Measure (FMFM) [43] will be used to evaluate motor function. The GMFM-66 includes sixty-six items in five categories (lying and rolling; sitting; crawling and kneeling; standing; walking, running, and jumping). The FMFM includes sixty-one items in 5 dimensions. For each item, the physiatrist describes it through language or demonstration before the test. Each item is scored on a four-point scale ranging from 0 to 3. Children are given three chances to try to complete the test item, and the best score is recorded. Higher scores represent better motor function.

The Faces Pain Scale-Revised (FPS-R) [44] will also be used to measure pain. The FPS-R consists of 6 facial expressions representing pain intensity from 0 to 10. Physiatrists judge the severity of pain by observing facial expressions such as frowning, deepening of the nasolabial groove, and opening the mouth.

Balance function will be measured by the interactive balance system (IBS; Tetrax Inc., Ramat Gan, Israel) [45]. This test involves having children maintain stability in standing position in eight postures for 30s each (stand on the force platform with eyes open/closed, stand on the foot pad with eyes open/closed, turn the head left/right with eyes closed, and lean the head forwards/back 30° with eyes closed). The susceptors on the soles of the feet measure the pressure on different parts of the plantar surface during the test and output a balance score with a maximum of 100. Higher scores indicate worse balance function.

The Barthel index will be used to measure subjects’ activities of daily living [46]. This scale contains 10 items, with each item scored on a scale of 0–5, 0–10, or 0–15. The items include eating, dressing, going up and down stairs, stool control, urination control, toilet use, transfer, flat walking, bathing, and grooming. The children’s ability to perform these tasks is scored. A higher score (out of a maximum of 100 points) reflects better functional independence.

Quality of life will be assessed by the Chinese version of the CP Quality of Life scale for children (CPQOL-child) [47] and the Pediatric Quality of Life Inventory Measurement Model: Cerebral Palsy Module (PedsQL-CP) [48]. The CPQOL includes a parent questionnaire and a self-report questionnaire. The parent questionnaire is suitable for children with CP aged 4–12 years. The self-report questionnaire is designed for children with CP aged 9–12 years. The PedsQL-CP is suitable for evaluating the quality of life of children with cerebral palsy, with a parent questionnaire designed for children aged 2–18 years, and a self-report questionnaire designed for children aged 5–18 years. Children who meet the age requirement of these questionnaires at baseline will be asked to complete the survey before and after treatment. The total score for each scale will be calculated after completion of the assessment.

#### Safety

Before the start of treatment, we will record the children’s history of seizures, syncope, headache, cognitive deficits, and behavioural disorders in detail. For children with adequate cognitive ability, subjective evaluation of treatment tolerance will be used to evaluate safety during and after treatment on a scale of 1 to 10, where 1 is the least pleasant and 10 is the most pleasant. If the score is less than 5 points, this reflects poor treatment tolerance, and the treatment will be stopped. For children with cognitive deficits, physiotherapists will observe the child’s expression during each treatment. According to the FPS-R, treatment should stop when there is frowning or deepening of the nasolabial groove on children’s faces. If mild adverse events occur (fatigue, local discomfort, headache), the treatment will be paused, the event will be recorded, and the treatment intensity will be adjusted. If severe adverse events (e.g., seizure) occur, the treatment will be stopped, emergency treatment will be provided, and the condition will be reported to the research leader.

### Statistical analysis

#### Main analysis

The R-FLACC scores before and after the intervention will be compared to determine the therapeutic effects in the three groups. Subjects in randomly assigned groups with baseline measurements will be enrolled in the analysis of primary outcomes using the following method.

 [1] All indicators will be analysed as continuous variables.

 [2] Boxplots will be used to detect outliers, the S‒W test will be used to test distribution normality, and Levene’s test will be used to test the homogeneity of variance.

 [3] A paired-sample t test will be used to analyse the difference in outcomes with data conforming to a normal distribution and homogeneous variance before and after the intervention. The Wilcoxon signed-rank test will be used to examine the difference before and after intervention for outcome measures that do not conform to a normal distribution or exhibit heterogenous variance.

 [4] The difference between the outcome measures of each group before and after the intervention will be calculated. An independent-sample t test will be used to analyse the difference between the groups if the differences are normally distributed and exhibit homogeneous variance; otherwise, the Wilcoxon signed-rank test will be used.

#### Secondary analysis

Data will be divided into an intention-to-treat analysis set and a safety analysis set. The intention-to-treat set will include subjects who were randomly assigned to groups and completed the baseline measurement. Subjects who received at least one intervention and underwent one safety assessment will be allocated to the safety analysis set. The change in secondary outcomes from before to after the intervention will be compared to determine the therapeutic effects in three groups using the intention-to-treat set through the same analytical method as the main analysis. The safety analysis will include counting the number of adverse events and the number of patients who drop out because of unbearable adverse events.

### Quality control and quality assurance

Outcome measurements will be collected by an experienced physiatrist, and physiotherapists in charge of the intervention will receive standardized training and assessments in advance. Before enrolment, an individual researcher will provide detailed information to the participants and their guardians to ensure that they understand the possible benefits of the treatment, thereby improving compliance.

A three-grade quality control system will be used for quality control: self-examinations from researchers, monthly examinations from group inspectors, and quarterly supervision from the research leader.

A clinical research unit (CRU) will be established to ensure the safety and validity of the data of this study. The CRU can terminate the trial early if a large number of adverse events occur or the data are proven to be invalid.

## Discussion

This protocol aims to explore the safety and effects of VR therapy combined with rTMS on pain and motor development in children with spastic CP. Considering comfort and the possibility of visual impairment in children with CP, we chose immersive VR training in this protocol, which is easier for physiotherapists to use than VR glasses or gamepads [49]. Kirton et al. [50] applied 1 Hz rTMS at 100% rMT for 20 min over the contralesional M1 for 8 days in children with hemiplegia. We chose a similar rTMS program since it resulted in significant motor improvement and was demonstrated to be safe and well tolerated.

The results of this protocol may reveal the additive effects of the combination of VR therapy and rTMS or the effects of rTMS only on pain management in spastic CP patients. Self-reported pain or observation of pain-related behaviours are the two main methods for pain assessment in spastic CP patients due to their potential impairments in cognition and verbal expression. Self-reported pain assessments are strongly recommended by guidelines for measuring the severity of pain, but it may not be reliable if the participants have cognitive deficits [51]. Tools for pain assessment, such as the FLACC, are widely used in individuals with cognitive or intellectual impairment [52]. The design of the FLACC was based on 89 postoperative children in the post-anaesthesia care unit aged 2 months to 7 years, and it was developed to evaluate pain in children who cannot express pain in words [53]. A previous study showed that the FLACC pain score was valid (Pearson’s correlation coefficients of 0.76 and 0.59 with the observational visual analogue scale score) and reliable (Cronbach’s alpha > 0.9) for assessing pain in children with CP; however, this study demonstrated insufficient validity in adult patients [54, 55].

In addition to the assessment of pain and motor development, we plan to utilize balance, activity of daily living, and quality of life assessments, which may reflect the possibility of these children to return to family and school. This study protocol may further bridge the gaps among pain, motor development, and functional independence, and the results may reveal the impact of pain on returning to society in children with CP.

Few adverse events have been reported in studies of rTMS utilizing high-frequency stimulation or low-frequency inhibition regimens. When rTMS is administered, the risk of seizures in children is very low (approximately 0.1%) [56]. Scalp discomfort at the irritation site and headache are mild and transient side effects of rTMS that may be reported by children and considered in the safety assessment in this protocol. VR therapy is considered a safe therapy, as a former meta-analysis showed that cybersickness and nausea are the most common side effects of VR therapy, with very low-certainty evidence, and no severe adverse events have been reported [17]. The treatment will be paused if cybersickness or nausea occur during the VR intervention.

This study protocol has some limitations. First, it may be difficult to blind the intervention practitioners (physiotherapists) during the intervention process because it may be easy for the physiotherapist to observe intervention methods in the immersive VR environment. Second, there may be bias in pain evaluations using the FLACC because of lower limb spasms in children with CP. To avoid the impact of lower limb spasms on the accuracy of pain assessment, pain assessment will be conducted by an experienced physiotherapist.

## Clinical implications

We expect to identify an effective method for simultaneously relieving pain in children with spastic CP and promoting motor function. Effective pain management can improve the quality of life of CP patients and is key to achieving better psychological development and overall prognosis. The results of this study may support future clinical practice and provide high-quality evidence regarding new approaches for pain management for children with CP. In addition, since central nervous system disorders frequently involve both pain and motor dysfunction [57], future studies should focus on the potential of combining VR therapy and rTMS in pain management in adults with neuropathic or musculoskeletal pain.

## Data Availability

Not applicable.

## Abbreviations

CP: Cerebral palsy
VR: Virtual reality
rTMS: Repetitive transcranial magnetic stimulation
GMFCS: Gross Motor Function Classification System
M1: Primary motor cortex
R-FLACC: Revised Face, Legs, Activity, Cry, and Consolability Scale
GMFM-66: 66-item version of the Gross Motor Function Measure
FMFM: Fine Motor Function Measure
C-CPQOL-Child: Chinese version of the Cerebral Palsy Quality of Life Scale for children
PedsQL-CP: Pediatric Quality of Life Inventory Measurement Model:Cerebral Palsy Module
rMT: Resting motor threshold
